# Collagen from Salted Jellyfish (*Rhopilema esculentum*): Structural Characterization, Emulsifying Properties and Wound Healing Potential

**DOI:** 10.3390/gels12070582

**Published:** 2026-07-01

**Authors:** Bing Hu, Zixin Zong, Lingyu Han, Ziang Yao, Jixin Yang, Ronggang Liu, Jijuan Cao, Saphwan Al-Assaf

**Affiliations:** 1 Key Lab of Biotechnology and Bioresources Utilization of Ministry of Education, College of Life Science, Dalian Minzu University, Dalian 116600, China; zongzixin11@163.com (Z.Z.); hanlingyu1001@126.com (L.H.); 20201460@dlnu.edu.cn (Z.Y.); lrg19950427@163.com (R.L.); 20191414@dlnu.edu.cn (J.C.); 2 Faculty of Social and Life Sciences, Wrexham University, Plas Coch, Mold Road, Wrexham LL11 2AW, UK; j.yang@glyndwr.ac.uk; 3 Hydrocolloids Research Centre, University of Chester, Chester CH1 4BJ, UK

**Keywords:** *Rhopilema esculentum*, emulsion stability, rheological property, wound healing, marine biomaterial

## Abstract

Jellyfish collagen, a sustainable and biocompatible marine biomaterial, holds great potential in food and biomedical applications. This study explores the emulsification properties and therapeutic potential of pepsin-soluble collagen derived from salt-preserved *Rhopilema esculentum* (RPSC). Structural analysis confirmed that RPSC retained an intact triple-helix structure with a denaturation temperature of approximately 36.0 °C and formed elastic gels at concentrations ≥1.5% (*w*/*w*). As an emulsion stabilizer, RPSC (2%, *w*/*w*) effectively stabilized oil-in-water emulsions with oil fractions up to 50%, forming viscoelastic networks that exhibited excellent centrifugal stability but limited freeze–thaw tolerance. The gel–sol transition occurred near 38.0 °C, consistent with the thermal denaturation of RPSC. As a wound healing promoter, RPSC showed no cytotoxicity and dose-dependently enhanced 3T3 fibroblast viability, migration, and SOD activity. Notably, RPSC downregulated TGF-β1 expression and suppressed endogenous type I collagen synthesis, indicating a scar-mitigating profile distinct from conventional pro-fibrotic collagen dressings. These findings establish RPSC as a bifunctional marine biomaterial for both emulsified food systems and regenerative wound dressings in the biomedical field.

## 1. Introduction

Jellyfish, widely found in temperate and tropical oceans, has been used in East Asian cuisine for centuries and typically preserved through salt curing for extended shelf life and easy transportation [1]. Although traditionally seen as a low-value salted food, its full potential remains underexplored. Fresh jellyfish is composed of 95–99% water, with a protein content of 3–5% and minerals at 1–2% [2]. Notably, its protein is almost entirely collagen, making jellyfish a promising source of collagen.

Collagen, a structural protein abundant in connective tissues, is valued for its gel-forming ability and biocompatibility, making it an essential resource in various industries. In food, it serves as an emulsifier, gelling agent, and stabilizer in products like dairy, beverages, and processed meats [3]. In biomedicine, collagen aids in wound healing [4], tissue regeneration [5], and drug delivery [6,7]. It is also used in cosmetics for its anti-aging properties and recently there has been significant interest in collagen for sport drinks [8,9].

In contrast to mammalian and fish-derived collagens, jellyfish-derived collagen exhibits several notable advantages. Jellyfish are characterized by their abundance and rapid proliferation; their frequent blooms are regarded as a marine ecological nuisance, yet the extraction of collagen from these organisms transforms an environmental challenge into a resource opportunity [3]. Jellyfish collagen is devoid of terrestrial pathogens, displays low immunogenicity, and lacks known major allergens. From a functional perspective, jellyfish collagen demonstrates a high degree of structural similarity to human collagen and possesses excellent biocompatibility [4]. These attributes position jellyfish collagen as a highly promising alternative material for biomedical and tissue engineering applications.

Among the known species of edible jellyfish, *Rhopilema esculentum* shows high availability and is thus an ideal candidate for further scientific investigation. While interest in jellyfish collagen has grown in recent years, most studies have focused on the collagen extracted from fresh jellyfish. However, in practical scenarios, the industrial processing of jellyfish typically involves salt-curing [10,11,12]. So far, studies focusing on the extraction and characterization of collagen from salt-cured jellyfish have been limited, leaving a critical gap in our understanding of its full potential.

Current research on emulsion stability predominantly focuses on Pickering emulsions and surfactant-based stabilization systems. Furthermore, given that the emulsification properties of collagen affect the overall emulsion stability, texture, and product quality of foods [13,14], a systematic investigation of the emulsifying properties of collagen derived from salt-cured *Rhopilema esculentum* is warranted. Such research could not only enhance its applications in the food industry but also lay the groundwork for innovative material development based on this understudied biopolymer. Given the promising biological properties of collagen, its functions extend beyond food production into the biomedical sector, and it is often used to promote wound healing.

While traditional dressings such as gauze provide fundamental wound coverage, they frequently lead to adhesion issues and inadequately manage exudate. In contrast, collagen, a key component of the extracellular matrix, serves as a bioactive alternative that enhances wound healing due to its biocompatibility, ability to support cell migration, and capacity to facilitate new tissue formation [15,16]. Given these beneficial properties, collagen derived from *Rhopilema esculentum* emerges as a promising candidate for the development of advanced, sustainable wound care materials, including bioactive scaffolds and dressings.

In this study, we employed a pepsin-assisted extraction method to isolate collagen from salt-cured *Rhopilema esculentum*. After confirming the key structural features (triple-helix integrity, thermal stability, and gelation behavior), we evaluated the emulsifying properties of this collagen by analyzing the stability of emulsions with varying oil volume fractions and testing their performance under different environmental stressors, including centrifugation, freeze–thaw cycles, and long-term storage. Finally, the wound healing potential of *Rhopilema esculentum* collagen was investigated by assessing its effects on mouse 3T3 fibroblast cell migration, adhesion, and the expression of key biomarkers, including superoxide dismutase (SOD) and transforming growth factor beta 1 (TGF-β1). This study provides valuable insights into the potential of *Rhopilema esculentum* as a sustainable marine source of high-value collagen. The findings of this research could possibly support the development of high-quality marine collagen-based food products and also highlight the potential for *Rhopilema esculentum* collagen in the fabrication of biofunctional materials for therapeutic applications, particularly wound healing.

## 2. Results and Discussion

### 2.1. Yield and Key Structural Properties of RPSC

#### 2.1.1. Yield and Fundamental Structural Properties

The RPSC yield obtained in this study was 1.15% (wet weight basis), comparable to previous reports for *Rhopilema esculentum* using pepsin-assisted extraction (0.28%) [17], with variations attributable to species, habitat, and processing methods.

Amino acid analysis (Table 1) revealed a characteristic collagen profile rich in glycine (21.50%), proline (7.09%), and hydroxyproline (6.20%). This profile is consistent with collagen’s central domain, which features conserved Gly-X-Y repeats essential for triple-helix formation [18]. Structurally, glycine is vital for forming the intra- and inter-chain hydrogen bonds that stabilize the structure [19,20]. Similar to other collagen sources, glycine was the most abundant amino acid in RPSC, accounting for 21.50% of the total amino acids. [21]. The imino acid content (proline and hydroxyproline) has been well established to be critical for maintaining collagen’s structural integrity and enhancing the stability of its triple-helix structure. These imino acids serve as key indicators of collagen stability and bioactivity and are commonly used as markers of collagen quality [22]. When compared to previously reported values, the hydroxyproline content of RPSC (6.20%) is notably higher than those reported for moon jellyfish collagen (4.3%) [23] and *R. pulmo* collagen collected from Goa Coast (4.82%) [24] but lower than that of sturgeon fish skin collagen (8.03%) [25]. Similarly, the proline content of RPSC (7.09%) is comparable to that of moon jellyfish collagen (7.8%) [23], higher than that of *R. pulmo* collagen collected from Goa Coast (2.97%) [24], and lower than that of sturgeon fish skin collagen (13.55%) [25]. These compositional differences are critically important for interpreting the rheological properties of RPSC. The moderate imino acid content of RPSC confers adequate thermal stability and gelation capacity for most food and biomedical applications. Thus, RPSC represents a functionally balanced marine collagen with practical advantages for industrial utilization.

**Figure 1:**
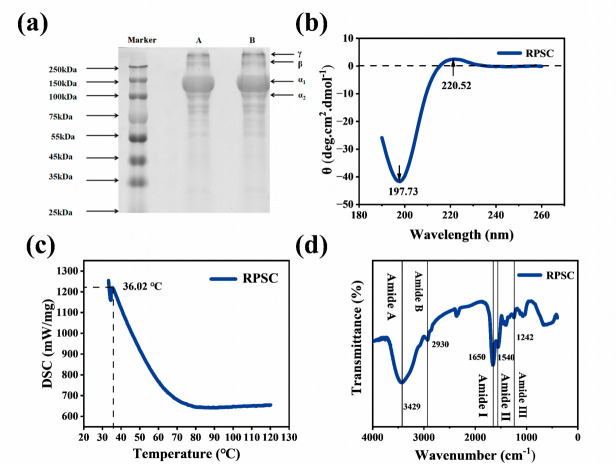
Structural properties of RPSC. (**a**) SDS-PAGE profile (A, RPSC without β-me; B, RPSC with β-me). (**b**) CD spectra. (**c**) DSC curve. (**d**) FTIR spectra.

Figure 1a shows the SDS-PAGE profile of jellyfish collagen, with distinct α1 (~130 kDa), α2 (~110 kDa), β, and γ chains (~250 kDa), consistent with previous reports [26]. Additional bands between 55–75 kDa suggest partial proteolytic cleavage of α chains during extraction due to incomplete protease inactivation [27]. These findings indicate that the subunit composition of RPSC matches the heterotrimeric type I collagen structure [α_1_(I)]_2_α_2_(I) [28]. Comparative analysis under reducing and non-reducing conditions revealed no discernible reduction in band intensity following β-mercaptoethanol (β-me) treatment, demonstrating the absence of disulfide bonds in RPSC [29,30]. Consistently, the trace amount of cysteine detected by amino acid analysis (1.44% of total protein, Table 1) likely originates from minor non-collagenous impurities or residual free amino acids, rather than from disulfide bonds within the RPSC triple helix. This biochemical property supports RPSC classification as type I collagen, whose triple-helical stability relies on hydrogen bonding and hydroxyproline-mediated interactions rather than disulfide crosslinking.

CD spectroscopy serves as an effective tool for elucidating the conformational structures of proteins [31]. Native collagen typically exhibits a characteristic triple-helical conformation, showing a positive absorption peak near 225 nm and a negative absorption band at approximately 197 nm. Upon complete denaturation, the positive peak disappears entirely while the negative band undergoes a redshift [32,33]. As demonstrated in Figure 1b, the CD spectrum of RPSC contains a negative peak at 197 nm as well as a positive signal near 220 nm, in line with the spectral signature of collagen’s triple helix conformation [34]. Importantly, the CD spectral features of RPSC are consistent with those reported for collagens extracted from fresh fish. Previous studies have shown that collagen from anglerfish [22] and skin of loach [35] typically displays a positive peak at 200–220 nm and a negative peak at 197–200 nm—almost identical to the profile observed for RPSC. This comparison confirms that RPSC sample retains its native triple-helical structure and has not suffered denaturation during processing or extraction. Thus, RPSC is structurally comparable to freshly extracted fish collagen, supporting its suitability as a reliable collagen source. These findings confirmed the retention of the triple-helical structure in RPSC. The triple-helical conformation of RPSC was further corroborated by comparison with a commercial benchmark. Faruqui et al. [36] reported that JellaGel^TM^, a commercial jellyfish collagen product, displays characteristic CD features of native collagen with a positive band at 215–220 nm and a negative band at 195–200 nm. The CD spectral profile of RPSC is nearly identical to that of this commercial product, confirming that RPSC retains its native triple-helical conformation and is structurally comparable to commercially available jellyfish collagen standards.

As shown in Figure 1c, the thermal denaturation temperature of RPSC was found to be approximately 36.0 °C. While this value is higher than those reported for many aquatic collagens, e.g., 25.6 °C for Scomber japonicus [37], 28 °C for Cyprinus carpio [38], and 32 °C for Oreochromis niloticus [39], it remains lower than mammalian collagen, e.g., 34–45 °C for porcine collagen [40]. This lower thermal stability, often linked to the habitat temperature, exhibits varying Td values. Notably, the thermal denaturation temperature of collagen is often positively correlated with the levels of hydroxyproline and proline in its structure. In fish collagen, Td values above 33 °C are already considered thermally resistant [41], suggesting RPSC may occupy a middle ground between typical aquatic and mammalian collagens. Notably, the observed Td of 36.0 °C may also be influenced by the salt-curing processing of jellyfish, in addition to species-specific characteristics. The salting process, which involves sequential treatments with sodium chloride and alum, may induce structural reorganization of collagen. Such processing-induced structural changes could potentially affect the thermal denaturation behavior of the extracted collagen.

FTIR spectroscopy provides critical information regarding the vibrational states of chemical bonds in a compound. As shown in Figure 1c, five characteristic amide bands were prominently observed in RPSC—Amide A, Amide B, Amide I, Amide II, and Amide III—with spectral characteristics similar to those observed in other collagen species, e.g., shark-skin [42], bovine corneas [43], and yellow belly pufferfish skin [44]. The Amide A band of RPSC appeared at 3429 cm^−1^, while the Amide B band was located at 2930 cm^−1^. The Amide I, II, and III bands were positioned at 1650 cm^−1^, 1540 cm^−1^, and 1242 cm^−1^, respectively. The Amide A band corresponded to N-H stretching vibrations, which have a characteristic range of 3400–3440 cm^−1^ and are observed when peptide NH groups participate in hydrogen bonding [45]. These observations confirmed the presence of hydrogen bonds between the NH groups and carbonyl groups within the peptide chains of jellyfish collagen. The Amide B band, detected at 2930 cm^−1^, was attributed to C-H stretching vibrations, which likely originated from the high abundance of proline and hydroxyproline residues in the side chains of jellyfish collagen. The Amide I band, primarily arising from C=O stretching vibrations, appeared at 1650 cm^−1^. This feature was characteristic of collagen molecules, indicating the well-preserved secondary structure of RPSC without the presence of any significant denaturation [46]. The Amide II band, associated with β-sheet structures, reflected contributions from NH bending vibrations and C-N stretching vibrations. Meanwhile, the Amide III band at 1242 cm^−1^, characteristic of α-helical conformations, originated from CN stretching vibrations coupled with in-plane NH bending [47,48]. Notably, the collective presence of Amide I, II, and III bands served as a diagnostic signature for the triple-helical structure characteristic of collagen proteins. These three amide bands exhibit strong intercorrelations [20,49,50], collectively providing critical insights into the structural integrity and conformational features of the collagen matrix. The FTIR spectral features of RPSC were also compared with those of commercially available jellyfish collagen. Paradiso et al. [51] successfully utilized Rhizostoma pulmo jellyfish collagen—the same species as RPSC—as a cell culture substrate, and reported FTIR characteristics consistent with those of native collagen. The RPSC FTIR profile (3283 cm^−1^, 2934 cm^−1^, 1647 cm^−1^, 1550 cm^−1^, and 1238 cm^−1^ for Amide A, Amide B, Amides I, II, and III, respectively) aligns closely with these reported values, indicating that RPSC is structurally comparable to commercial-grade jellyfish collagen standards.

#### 2.1.2. Microstructure and Rheological Behavior of RPSC

**Figure 2:**
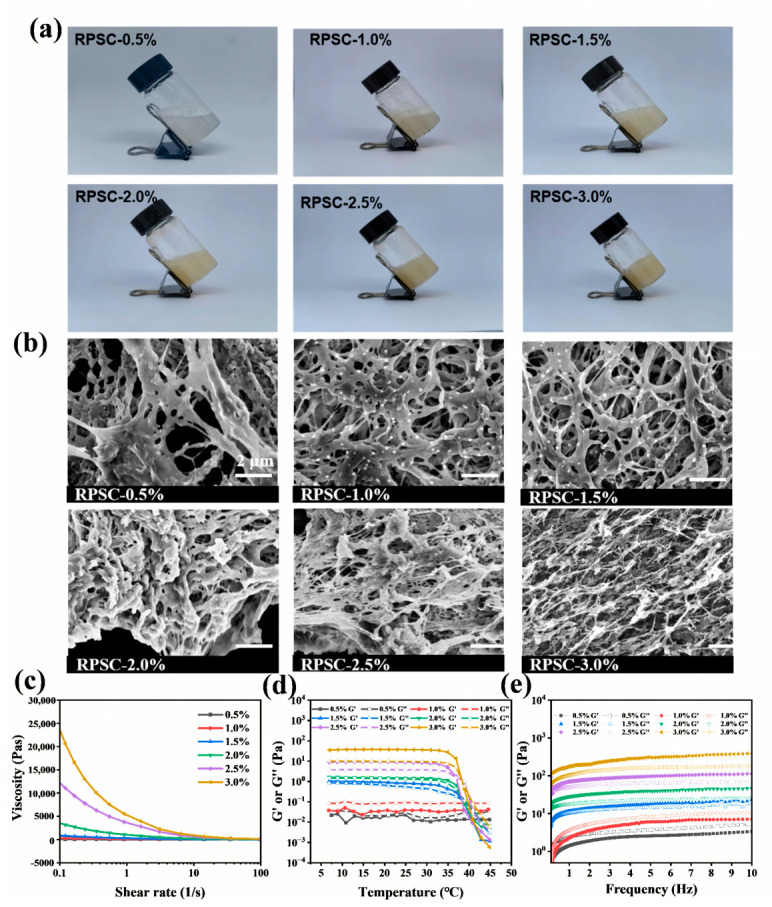
(**a**) Appearance and (**b**) SEM images of RPSC gels. (**c**–**e**) Rheological behavior of RPSC gels. Apparent viscosity (**c**), Temperature scanning curves (**d**) and Storage modulus G′ and loss modulus G″ versus frequency (**e**).

As the RPSC concentration increased, the viscosity of the system rose significantly and gradually exhibited a typical gel-like structure (Figure 2a). This phenomenon is primarily attributed to the physical crosslinking behavior of collagen molecules with increasing concentration. At low PSC concentrations, collagen molecules remained dispersed in aqueous solution with weak intermolecular interactions, whereas increasing the concentration enhanced molecular contact probability, promoting the formation of a three-dimensional network through non-covalent interactions (e.g., hydrogen bonds, hydrophobic interactions, and electrostatic attractions), thereby facilitating the sol-to-gel transition. Scanning electron microscopy (Figure 2b) revealed that the jellyfish collagen gels exhibited a typical porous network morphology. As PSC concentration increased, the gel network gradually evolved from a loose, irregular pore structure to a uniform and dense three-dimensional network, attributed to enhanced intermolecular interactions among collagen molecular chains, leading to more crosslinking sites and progressive network refinement.

Rheological measurements further supported this concentration-dependent network formation. All RPSC solutions exhibited shear-thinning behavior, where viscosity decreased with increasing shear rates due to disruption of hydrogen bonds and the collagen network (Figure 2c). During temperature sweeps (Figure 2d), RPSC solutions at intermediate-to-high concentrations (1.5–3%) exhibited stable, elastic gel behavior (G′ > G″) at low temperatures (5–32 °C), with both moduli increasing non-linearly with concentration due to enhanced intermolecular cross-linking. A critical transition occurred near 32 °C, marked by a decrease in moduli indicating triple-helix unfolding, and the gel–sol transition completed at 38 ± 0.5 °C (G′ = G″), above which viscous behavior (G″ > G′) dominated. Notably, this transition temperature was concentration-independent, reflecting intrinsic stability of intramolecular structures. The thermal gel–sol transition of collagen is mechanistically rooted in the thermal denaturation of its triple-helical structure (Figure 1c). Gel formation is exclusively enabled by the intact native triple helix—a conformation stabilized synergistically by intramolecular hydrogen bonds, intermolecular hydrophobic interactions, and the stereospecific conformational constraint imposed by hydroxyproline residues. Upon heating, thermal energy selectively destabilizes the hydrogen-bonding network intrinsic to the triple helix, triggering localized helix unwinding. Further temperature elevation promotes progressive, cooperative dissociation of the triple-helical architecture, driving the transition of collagen molecules from a rigid, rod-like conformation to a flexible, disordered random coil. This structural collapse abolishes the molecular prerequisites for physical crosslinking: in the gel phase, collagen monomers assemble into a percolating three-dimensional network via chain entanglement and reversible weak interactions—including hydrogen bonding, hydrophobic contacts, and van der Waals forces—thereby immobilizing water within the porous matrix. Thermal denaturation erodes the density and spatial complementarity of interaction interfaces, undermining network connectivity and leading to structural disintegration and water expulsion. Macroscopically, this manifests as a sharp, thermoreversible gel–sol transition. Thus, the thermal gel–sol transition is not merely correlated with but is a direct macroscopic consequence of collagen’s molecular denaturation; accordingly, the gel–sol transition temperature exhibits a robust positive correlation with the thermal denaturation midpoint temperature. In contrast, the 0.5% and 1% solution showed viscous dominance (G″ > G′) across all temperatures, resulting from a discontinuous network with insufficient cross-links. Dynamic frequency sweeps (Figure 2e) confirmed that a continuous three-dimensional network formed at RPSC concentrations ≥1.5%, as evidenced by solid-like behavior (G′ > G″), whereas the 0.5% and 1% solution remained viscous-dominated. Both moduli increased with concentration, reflecting enhanced network density. Collectively, these results demonstrate that increasing PSC concentration promotes a denser, more uniform gel network, which translates into improved viscoelastic properties and gel strength.

### 2.2. Emulsification Properties of RPSC

#### 2.2.1. Visual Appearance and Microstructure

**Figure 3:**
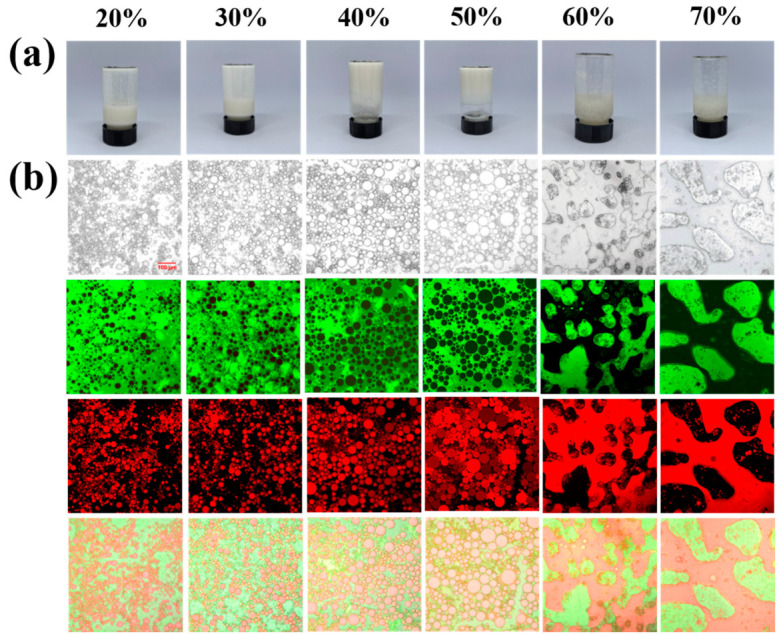
Visual appearance (**a**) and microscopic images (**b**) of RPSC-stabilized emulsions with different oil volume fractions.

Figure 3a shows that the self-supporting structure of RPSC-stabilized emulsions strengthened with increasing oil fraction up to 50%, beyond which phase separation occurred. Corresponding CLSM images (Figure 3b) revealed uniformly distributed and progressively larger lipid droplets at 20–50% oil, whereas based on visual observation and supported by CLSM image analysis, distinct demulsification was observed at 60–70% oil [52]. Specifically, after allowing the samples to stand for 30 min, the degree of demulsification was assessed by visually comparing the separation of oil and aqueous phases across different oil fractions, with CLSM images further confirming phase separation at 60–70% oil. This microstructural transition can be explained by interfacial effects. Higher oil fractions provided more interfacial area, promoting RPSC adsorption, enhancing droplet interactions to form a tightly packed, continuous 3D network at 50% oil, and resulting in a non-flowing gel [53]. However, once the oil fraction exceeded 60%, the fixed 2% collagen concentration was insufficient for complete interface coverage, which led to droplet coalescence and the eventual collapse of the emulsion structure. These findings highlight a nonlinear relationship between oil fraction and stability, underscoring the importance of balancing the emulsifier concentration and dispersed phase volume.

#### 2.2.2. Rheological Properties of Emulsions

**Figure 4:**
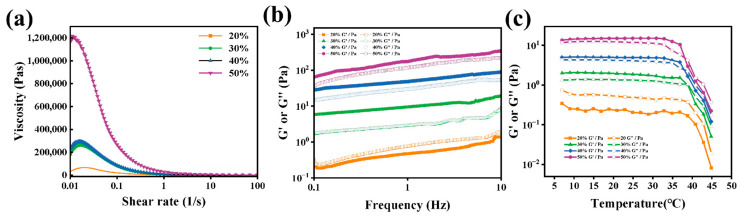
Rheological characterization of RPSC-stabilized emulsions with varying oil fractions. (**a**) Apparent viscosity. (**b**) Storage modulus G′ and loss modulus G″ versus frequency. (**c**) Temperature scanning curves.

Rheological analysis was conducted on collagen-based emulsions with oil fractions of 20–50%, as higher fractions (60–70%) resulted in demulsification (Figure 3). Within the stable range, emulsion viscosity increased with oil content (Figure 4a), indicating enhanced interactions among droplets and a denser network. Frequency sweeps (Figure 4b) revealed predominantly elastic behavior (G′ > G′′) in all except the 20% oil emulsion, which was viscous-dominant (G′′ > G′). Both moduli increased with oil content, reflecting stronger molecular interactions and gel network formation. Moreover, the temperature-dependent rheological profiles (Figure 4c) of the emulsions were similar to those shown in Figure 2d. Figure 4c showed persistent elastic dominance (G′ > G′′) below 38 °C for emulsions with ≥30% oil, while all samples underwent a sharp modulus decrease near 38 °C, with higher oil formulations exhibiting a clear gel–sol transition (G′ = G′′). This suggests the thermal collapse of the colloidal network, likely due to collagen denaturation. The results indicate that RPSC forms an elastic, stabilizing network within the emulsion, whose strength increases with oil fraction up to the critical point but is disrupted by elevated temperatures.

#### 2.2.3. Centrifugal Stability

**Figure 5:**
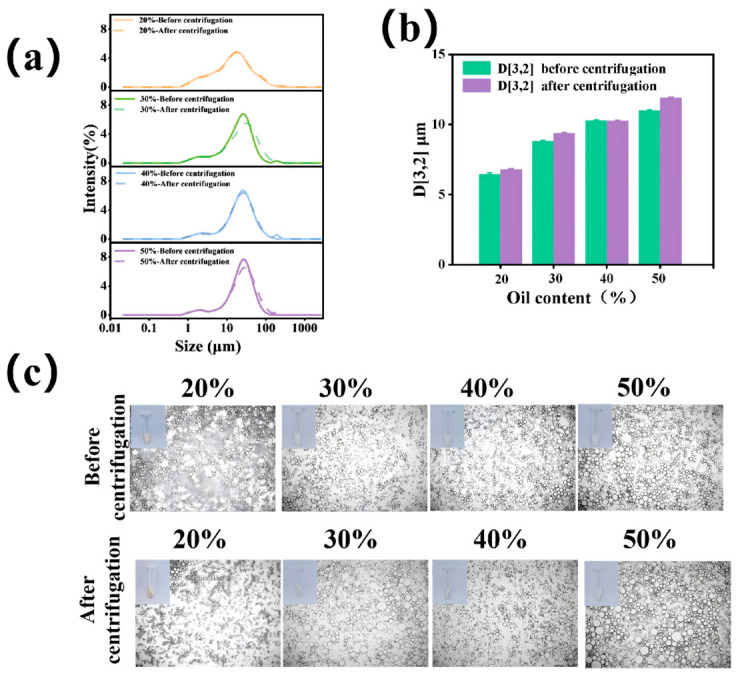
Oil droplet size distribution before and after centrifugation (**a**); average droplet size (**b**); visual appearance and microscopic images of RPSC-stabilized emulsions with different oil volume fractions before and after centrifugation (**c**).

Stability is a critical indicator of the scientific and practical value of emulsion-based products. Centrifugal stability analysis is commonly employed to assess the structural changes in emulsions during transportation [54]. As illustrated in Figure 5a,b, before centrifugation, the droplet size of the emulsions progressively increased with an increase in the oil volume fraction when the aqueous-phase protein concentration was fixed. This trend correlated with reduced protein availability per unit oil phase volume and diminished interfacial coverage. The surface-weighted mean diameter D [3,2] was selected as the primary size descriptor because it is inversely proportional to the total interfacial area per unit volume. In emulsion systems where stability is governed by interfacial protein coverage and surface-driven phenomena. Following centrifugation, all emulsions exhibited a slight increase in droplet size. This increase was likely due to the centrifugal force, which causes the particles in the emulsion to come close to each other and aggregate or coalesce, forming larger particles. Microscopy images (Figure 5c) further supported the above hypothesis. Additionally, as demonstrated in Figure 5a the centrifuged emulsions maintained relatively stable structures, with no significant shift in the particle size distribution. This phenomenon could be attributed to the capacity of the three-dimensional droplet network to dissipate centrifugal stress via the elastic deformation of interfacial proteins, thereby suppressing droplet coalescence. Visually, all emulsions displayed creaming post-centrifugation, with the intensity of phase separation diminishing as the oil phase fraction increased (Figure 5c). Higher oil volume fractions are believed to enhance the viscoelasticity of emulsions, thereby facilitating the formation of more stable three-dimensional network structures [55]. Notably, the reinforced networks demonstrate improved resistance against centrifugal shear forces, thereby preventing demulsification [56]. These findings collectively demonstrate the critical role of the oil volume fraction in optimizing the centrifugal stability of emulsions through structural reinforcement mechanisms.

#### 2.2.4. Freeze–Thaw Stability

**Figure 6:**
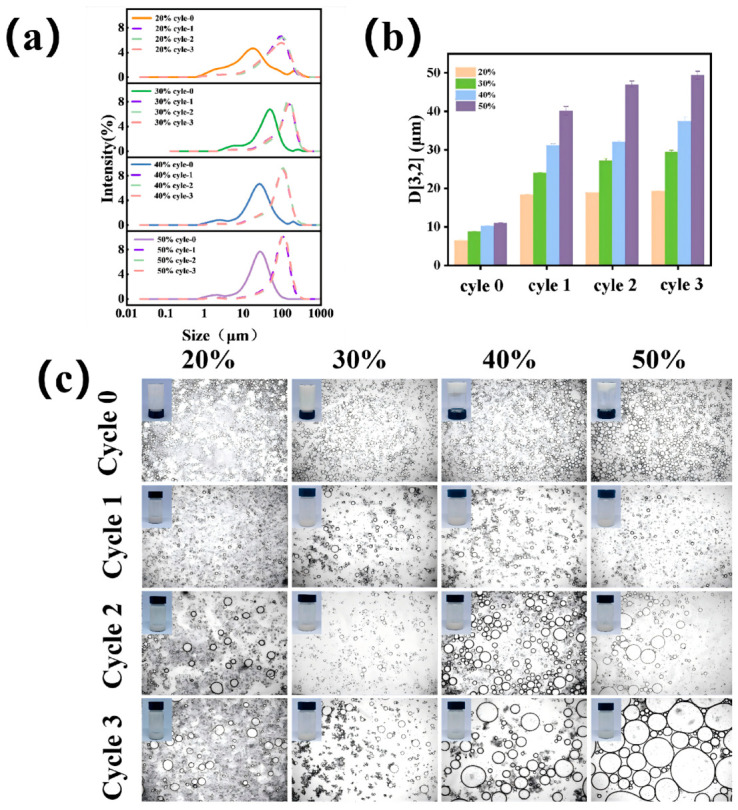
Oil droplet size distribution before and after three freeze–thaw cycles (**a**); average droplet size (**b**); visual appearance and microscopic images of RPSC-stabilized emulsions with different oil volume fractions before and after three freeze–thaw cycles (**c**).

Figure 6a shows that as the number of freeze–thaw cycles increased, the particle size distribution of the emulsions shifted toward larger particle sizes, with the surface mean diameter (D [3,2]) increasing (Figure 6b). This particle size evolution clearly demonstrates the limited freeze–thaw resistance of RPSC-stabilized emulsions, indicating that the emulsion microstructure is susceptible to irreversible structural damage under alternating low- and high-temperature conditions. This could be due to temperature fluctuations, which caused the emulsion interface to rupture, leading to droplet coalescence. The damage caused by each freeze–thaw cycle accumulated, resulting in a gradual increase in particle size [57]. The visual appearance of the emulsions indicated that during the freeze–thaw cycles, the emulsions exhibited needle-like and dendritic patterns with accompanying phase separation. Oil droplets were observed on the surface of the emulsion after freeze–thaw treatment (Figure 6c). It is speculated that this was caused by the formation of ice crystals when the aqueous phase froze, which typically grew in a dendritic fashion at low temperatures. The growth of ice crystals induced mechanical stress within the emulsion’s structure, irreversibly puncturing the interface, disrupting the emulsifying layer, and causing droplet rupture and oil separation [58]. Furthermore, the freeze–thaw cycle may also compromise the structural integrity of the emulsifier itself (RPSC). Repeated freezing and thawing deform the structure of the adsorbed protein layer. This structural change reduces its interfacial activity and weakens its ability to stabilize emulsions. This is likely due to strengthening the hydrophobic interactions leading to aggregation and thus reducing the surface coverage of the oil droplet [59]. Phase separation occurs due to the volume expansion of ice crystals, compressing the internal structure of the emulsion and disrupting the stability of the emulsifying agent and the oil-water interface. This volume expansion generates compressive forces that further destabilize the already weakened interface [60]. In this study, microscopy images (Figure 6c) demonstrated that as the oil volume fraction increased, the emulsion droplets showed progressive aggregation into larger droplets following the freeze–thaw cycles. These observations were consistent with the appearance of the emulsions. In systems with higher oil volume fractions, the water phase was reduced, limiting ice crystal growth. This could result in the formation of sharper crystals, exacerbating the mechanical puncture effect and causing droplet enlargement and oil separation.

#### 2.2.5. Storage Stability

**Figure 7:**
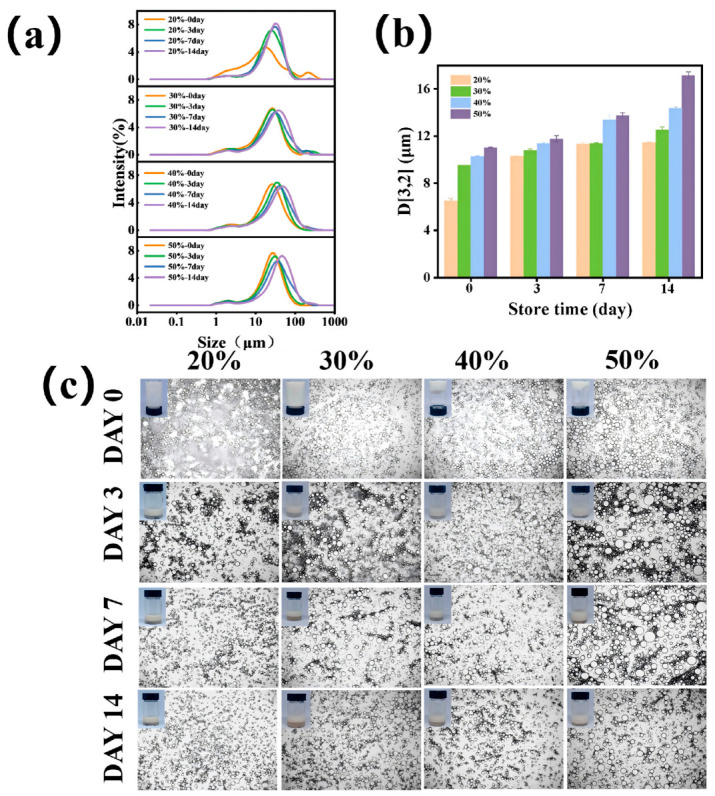
Oil droplet size distribution after 14 days of storage at 50 °C (**a**); average droplet size (**b**); visual appearance and microscopic images of RPSC-stabilized emulsions with different oil volume fractions after storage at 50 °C for 14 days (**c**).

During the 14-day storage at 50 °C, the rightward shift in particle size distribution (Figure 7a) and the corresponding increase in D [3,2] values over time confirmed progressive droplet growth (Figure 7b). The visual appearance of the emulsions during storage is shown in Figure 7c. After 3 days of storage at 50 °C, all emulsions exhibited creaming, with the creaming severity diminishing progressively as the oil phase fraction increased from 20% to 50%. This reduction was attributed to the formation of a denser droplet network structure in the presence of higher oil fractions, which physically impeded phase separation [61]. The microstructure of the emulsion (Figure 7c) further supported this hypothesis. These results collectively demonstrated that RPSC emulsions with an oil volume fraction of 50% exhibited optimal storage stability. Elevated storage temperatures (50 °C) significantly compromised emulsion stability by accelerating droplet aggregation and coalescence while simultaneously promoting phase separation, which disrupted the structural integrity of the system.

#### 2.2.6. Mechanistic Analysis of RPSC Emulsions

The excellent stabilization performance of RPSC on oil-in-water emulsions can be explained by the synergistic effect of interfacial adsorption and viscoelastic network formation. As an amphiphilic biopolymer, RPSC molecules can rapidly adsorb and arrange orderly at the oil-water interface, effectively reducing the interfacial tension of the emulsion system. The densely packed RPSC interfacial layer provides strong steric repulsion, which prevents the mutual contact and aggregation of dispersed oil droplets and inhibits emulsion destabilization caused by flocculation and coalescence. In addition to the interfacial protective effect, the accumulated RPSC in the continuous aqueous phase can further self-assemble to form a continuous and rigid viscoelastic gel network. This three-dimensional network structure physically immobilizes oil droplets within the aqueous matrix, effectively restricting droplet migration, creaming, and sedimentation. The combined effects of interfacial film protection and bulk viscoelastic network confinement endow the RPSC-stabilized emulsion with outstanding structural stability, which further verifies that RPSC is an efficient and reliable natural emulsifier for constructing stable oil-in-water emulsion systems. Our results are consistent with those reported on gum Arabic where it shown that the elasticity of the interface significantly enhances the emulsion stability of oil in water emulsion [62]. Nakauma et al. [63] reported the emulsifying properties of sugar beet pectin, soybean soluble polysaccharide, and gum Arabic at 1.5%, 4.0% and 10% respectively and MCT was the oil phase at 15%. They reported the number of molecules adsorbed onto the oil droplets is in the order SBP < SSPS < GA. The results obtained in this study compare favourably with those reported for sugar beet pectin, soybean soluble polysaccharide and gum Arabic due to RPSC’s ability for surface coverage of the oil droplet at significantly lower concentrations.

### 2.3. Wound Healing Potential of RPSC

#### 2.3.1. Assessment of Fibroblast Viability

**Figure 8:**
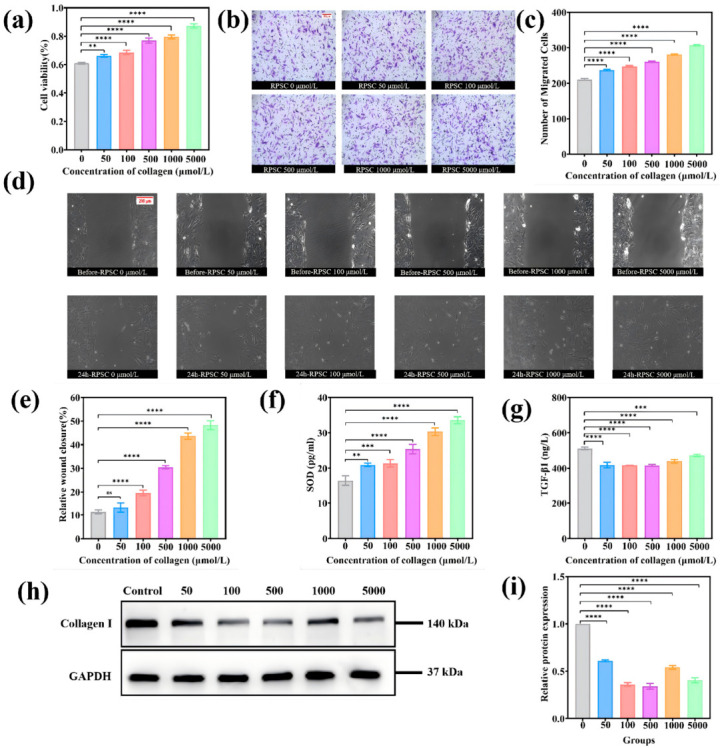
In vitro assays in 3T3 cells. (**a**) Viability of 3T3 cells after 24 h of treatment with different RPSC concentrations. (**b**) Microscopy images of Transwell assay. (**c**) Migration ability of 3T3 cells quantified using the Transwell assay. (**d**) Microscopy images showing wound closure in the wound healing assay. (**e**) Wound healing rates and percentage of the scratch area after 0 and 24 h of treatment with different RPSC concentrations. (**f**) SOD activity in 3T3 cells examined using ELISA. (**g**) TGF-β1 levels in 3T3 cells examined using ELISA. (**h**,**i**) Relative expression level of the Collagen I protein in different groups (**h**) examined using a Western blot assay (**i**). Data are presented as the mean ± SD. ns: not significant, * *p* < 0.05, ** *p* < 0.01, *** *p* < 0.001, **** *p* < 0.0001.

The viability of cells treated with RPSC was measured using the MTT assay. Figure 8a demonstrates that RPSC exhibited no cytotoxicity in 3T3 cells. As the concentration of RPSC increased, cell viability also rose, with 5000 µmol/L RPSC increasing cell survival by 59.86% compared to the control (**** *p* < 0.0001), indicating that RPSC promotes cell proliferation. It is speculated that this is due to RPSC promoting fibroblast proliferation by activating the PI3K/Akt/mTOR signaling pathway [53]. These findings confirmed its biocompatibility [26].

#### 2.3.2. Evaluation of Fibroblast Migration

Cell migration also plays a critical role at all stages of wound healing and is indispensable for the repair of damaged tissues and the regeneration of skin [64]. The migratory capacity of 3T3 cells (fibroblasts), which are closely involved in wound healing, was evaluated through Transwell assays (Figure 8b,c) and scratch wound healing assays (Figure 8d,e). Compared to the control group, the RPSC treatment groups showed enhanced cell migration rates, with 5000 µmol/L RPSC significantly increasing the number of migrated cells (**** *p* < 0.0001), and these enhancements became increasingly pronounced at higher concentrations of RPSC. At 24 h post-treatment with 5000 μmol/L RPSC, the scratch area of treated cells reached 48.25%, which was significantly higher than that in the control group (11.32%) (**** *p* < 0.0001) (Figure 8e). Similarly, the Transwell assays confirmed that RPSC promotes cell migration (Figure 8c).

#### 2.3.3. Measurement of SOD Activity

SOD catalyzes the dismutation of superoxide radicals in organisms, protecting cells and exerting anti-aging, antitumor, and anti-inflammatory effects [65]. Figure 8f shows that treatment with varying concentrations of RPSC dose-dependently increased SOD expression with significant elevation versus the control (**** *p* < 0.0001), suggesting that collagen may enhance cellular antioxidant capacity. This increase in SOD expression likely mitigated the reactive oxygen species (ROS)-induced damage to peri-wound tissues, thereby alleviating oxidative stress during the inflammatory phase [66]. Given that oxidative stress is a key driver of tissue damage, ROS inhibition could shorten the inflammatory phase and accelerate progression to the proliferative phase [67].

#### 2.3.4. Analysis of TGF-β1 and Type I Collagen Expression Levels

Notably, the levels of TGF-β, a multifunctional cytokine typically upregulated during inflammation [68,69], were also found to be significantly reduced following RPSC treatment (Figure 8g). Further, WB assays revealed that type I collagen expression also markedly decreased following RPSC treatment (Figure 8h,i). Mechanistically, the downregulation of TGF-β1 and the subsequent inhibition of type I collagen synthesis are key events that mitigate scar formation. During the proliferative phase of wound healing, fibroblasts are activated into myofibroblasts, which participate in the synthesis and remodeling of the extracellular matrix. However, excessively activated myofibroblasts often lead to abnormal collagen deposition, ultimately resulting in scar formation. Persistent high expression of TGF-β1 continuously activates fibroblast-myofibroblast transdifferentiation, leading to irreversible contraction of wound tissues, disordered extracellular matrix (ECM) arrangement, and excessive accumulation of fibrous proteins, which further induces tissue fibrosis and pathological scar hyperplasia. [70]. It is hypothesized that the TGF-β/SMAD signaling pathway may be involved in the tissue regeneration process. Smad7 (Mothers against decapentaplegic homolog 7) is an inhibitory SMAD protein that serves as a negative feedback regulator of the TGF-β/SMAD pathway. RPSC effectively activates the negative feedback loop of the TGF-β/SMAD signaling pathway by upregulating Smad7 expression. High concentrations of RPSC may enhance the negative feedback regulation of the TGF-β/SMAD pathway, potentially by inducing the expression of the inhibitory Smad7. As an intracellular antagonist, Smad7 effectively suppresses the phosphorylation and signaling transduction of Smad2/Smad3, thereby negatively feedback-inhibiting TGF-β1 expression and its pro-fibrotic function. Specifically, the increased Smad7 blocks the transduction of upstream TGF-β1 profibrotic signals, inhibits the continuous activation of downstream fibrotic cascades, and ultimately reduces the abnormal synthesis and accumulation of type I collagen. This targeted regulation effectively avoids ECM over-remodeling and abnormal tissue contraction during wound repair. Through this mechanism, RPSC regulates the TGF-β/SMAD signaling pathway and downregulates type I collagen expression, thus preventing excessive deposition of endogenous collagen and promoting scarless skin wound healing. This is significant because, during later stages of wound healing, excess fibrosis can lead to pathological scarring (e.g., hypertrophic scars or keloids), while TGF-β1 may reduce tissue remodeling quality.

## 3. Conclusions

In this study, we isolated pepsin-soluble collagen (RPSC) from processed *Rhopilema esculentum* and established its dual functionality as a structural stabilizer and a therapeutic agent. Unlike collagens extracted from unprocessed fish (e.g., skin, scale, and bone of fresh or frozen fish), which represent the more conventional marine collagen sources, RPSC is derived from salt-cured jellyfish—an industrially processed product and more commonly available. Structurally, both RPSC and unprocessed fish collagens are classified as type I collagens with a heterotrimeric [α1(I)]_2_α2(I) composition. The thermal denaturation temperature (Td) of RPSC (36.0 °C) is higher than that of cold-water fish collagens. The salting process has been shown to induce collagen fiber aggregation and structural reorganization, which may contribute to the observed Td. Functionally, both RPSC and unprocessed fish collagens exhibit biocompatibility and promote cell migration. However, a distinctive feature of RPSC is its intrinsic anti-fibrotic activity—upregulating SOD while downregulating TGF-β1 and endogenous type I collagen—which is not commonly reported for unprocessed fish collagens. As an emulsion stabilizer, RPSC formed robust viscoelastic networks at oil-phase fractions ≤50%, demonstrating excellent resistance to centrifugation. This defines its optimal application window for creating stable colloidal systems in food and cosmetic products. Its defined limitations (freeze–thaw and long-term thermal instability) are not merely drawbacks but provide critical parameters for designing effective formulations that leverage its strengths. As a wound-healing promoter, RPSC displayed exceptional biocompatibility, actively stimulating fibroblast proliferation and migration. Its unique value lies in a dual-action mechanism that works by enhancing tissue repair through the upregulation of antioxidant SOD while concurrently suppressing excessive fibrosis via the downregulation of TGF-β1 and endogenous type I collagen. This anti-fibrotic effect positions RPSC as a superior alternative to conventional mammalian collagens, which often lack such inherent scar-mitigating properties. By bridging the gap between marine resource utilization and biomedical innovation, this study suggests *Rhopilema esculentum* collagen as a versatile and sustainable biomaterial. Future efforts will focus on optimizing extraction yields, improving emulsion stability under industrial stress, and validating these promising in vitro results in vivo to advance translational applications, quantitatively comparing RPSC with common food emulsifiers and commercially available collagen preparations, as well as further investigating the molecular mechanisms by which RPSC reduces scar formation, particularly the detailed signaling pathways involved in TGF-β1 downregulation and collagen synthesis inhibition.

## 4. Materials and Methods

### 4.1. Materials and Reagents

Salted jellyfish was obtained from the Dongshan market in Dalian, China. Acetic acid, KBr, and sodium chloride were purchased from Tianjin Komio Chemical Reagent Co., Ltd. (Tianjin, China). Pepsin was acquired from Sinopharm Chemical Reagent Co., Ltd. (Shanghai, China). Medium-chain triglycerides (MCT) were acquired from Shanghai Yuanye BioTechnology Co., Ltd. (Shanghai, China). 3T3 fibroblast cells, paraformaldehyde, crystal violet staining solution, and BCA protein assay kit were purchased from Beyotime Co., Ltd. (Shanghai, China). 3T3-L1 Cell Complete Medium was acquired from Procell Co., Ltd. (Wuhan, China). The MTT Cell Proliferation and Cytotoxicity Assay Kit was provided by Solarbio Life Sciences Co., Ltd. (Beijing, China). The Mouse Superoxide Dismutase (SOD) ELISA Kit was acquired from Cusabio Technology Co., Ltd. (Wuhan, China). The TGF-β1 ELISA Kit was provided by the Nanjing Jiancheng Bioengineering Institute (Nanjing, China). The chemiluminescent detection substrate was purchased from Millipore Co., Ltd. (Billerica, MA, USA). GAPDH antibody and collagen type I polyclonal antibody were obtained from Proteintech Group Co., Ltd. (Wuhan, China). Goat anti-mouse IgG (H&L) secondary antibody and goat anti-rabbit IgG (H&L) secondary antibody were acquired from Thermo Co., Ltd. (Massachusetts, MA, USA). All the chemicals and reagents were of analytical grade.

### 4.2. Preparation and Yield of RPSC

To ensure consistency, all jellyfish samples were prepared using the traditional triple-alum processing method. This procedure involved sequential treatments with a mixture of NaCl and alum (KAl(SO4)_2_·12H_2_O), followed by dehydration. Salted jellyfish samples were thoroughly rinsed and cut into small pieces. To eliminate potential interference from salt ions, the completeness of rinsing was confirmed by a silver nitrate test before proceeding. After delipidation in anhydrous ethanol (1:10, *w*/*v*) for 24 h and rinsing until odor-free, the samples were swollen in 0.05 M acetic acid (1:15, *w*/*v*) for 12 h. The homogenate was then subjected to enzymatic hydrolysis with 0.5 M acetic acid (1:5, *w*/*v*) and pepsin (4%, *w*/*w*) for 48 h at 4 °C, at a final pH of approximately 2.5–3.0 [35,71]. Following centrifugation, the supernatant was adjusted to pH 7.0 with 1 M NaOH to inactivate the enzyme. For further purification of collagen, 2 M NaCl solution was added to the neutralized supernatant for salting-out precipitation to separate collagen from miscellaneous proteins and small molecular impurities. The precipitated crude collagen was collected by centrifugation, redissolved in 0.1 M acetic acid solution, and then subjected to stepwise dialysis purification at 4 °C. The dialysis was first performed with 0.1 M acetic acid for 48 h (the dialysate was replaced every 4 h to fully remove salt ions and small molecular impurities), followed by continuous dialysis with deionized water for another 48 h with the same dialysate replacement frequency to remove residual acetic acid and trace impurities [26,72]. The final solution was lyophilized to obtain *Rhopilema esculentum* pepsin-soluble collagen (RPSC) [73].

The extraction yield of RPSC was calculated based on the dry weight of purified lyophilized collagen and the initial dry weight of jellyfish raw materials. All extraction and purification operations were performed in triplicate independent parallel experiments under consistent temperature, solution concentration, solid–liquid ratio, reaction time and pH conditions. The specific calculation formula was as follows:

Yield (%) = Weight of dried collagen (g)/Weight of jellyfish used (g) × 100%

### 4.3. Key Physicochemical Characterization of RPSC

#### 4.3.1. Amino Acid Composition

Samples were hydrolyzed with 6 M HCl at 110 °C for 24 h, and amino acid contents (g/100 g) were quantified using an amino acid analyzer (L-8900, Hitachi, Tokyo, Japan).

#### 4.3.2. SDS-PAGE Analysis

SDS-PAGE was performed according to Laemmli [74]. RPSC solutions were mixed with loading buffer, with or without β-mercaptoethanol (β-me), heated at 95 °C for 5 min, and centrifuged. Supernatants were loaded onto an electrophoretic separation system. Electrophoresis was run at 80 V through the stacking gel and 120 V through the resolving gel until the dye front reached the bottom.

#### 4.3.3. CD Analysis

The secondary structure of RPSC was analyzed using a J-1500 spectropolarimeter (JASCO, Tokyo, Japan). Triplicate scans were averaged from 190 to 260 nm at 50 nm/min with 1 nm bandwidth (25 °C).

#### 4.3.4. Thermal Denaturation Temperature (Td)

DSC analysis scanned 5 mg samples in sealed aluminum crucibles from 30 °C to 120 °C at 2 °C/min under N_2_ [19]. The samples were tested a minimum of three times to guarantee the absence of deviation.

#### 4.3.5. Thermal Denaturation Temperature (FTIR)

The FTIR spectrum of RPSC was acquired using a Scimitar 2000 FTIR spectrometer (Thermo Electron, Madison, WI, USA). Samples were prepared by homogeneously mixing 5 mg of RPSC with 500 mg of dried KBr. The mixture was ground into fine powder and pressed into pellets. Spectral analysis was conducted at 25 °C over a wavenumber range of 400–4000 cm^−1^, with 64 cumulative scans undertaken at a resolution of 4 cm^−1^ [75].

#### 4.3.6. Microstructure of RPSC Gels

RPSC was dissolved in pure water to prepare RPSC solutions with concentrations of 0.5%, 1.0%, 1.5%, 2.0%, 2.5%, and 3.0% (*w*/*v*). Each collagen gel was cut into small pieces, fixed with 0.1 mol/L phosphate buffer (pH 6.8) and 2.5% (*v*/*v*) glutaraldehyde, and then dehydrated sequentially with 50%, 70%, 90%, and 100% (*v*/*v*) ethanol. The dehydrated gels were freeze-dried. The freeze-dried gel samples were cut into thin sections, mounted onto scanning electron microscope stubs, sputter-coated with gold, and observed at an accelerating voltage of 5.0 kV with a magnification of 100× [76].

#### 4.3.7. Rheological Properties of RPSC Gels

Rheological properties were measured using a Haake rheometer (MARS 40, Thermo Fisher, Waltham, MA, USA). Apparent viscosity was measured at 25 °C with shear rates from 0.01/s to 100.00/s. Storage (G′) and loss (G″) moduli were determined by frequency sweep (0.1–10 Hz) at 0.5% strain. Temperature sweeps from 5 °C to 45 °C at 1.0 °C/min were conducted to monitor G’ and G”. The gelation point was defined as the temperature where G’ equaled G” during heating [77].

### 4.4. Emulsion Preparation and Characterization

#### 4.4.1. Emulsion Preparation

Emulsions were prepared by homogenizing a 2.0% (wt%) RPSC solution with MCT at volume fractions ranging from 20% to 70% (three cycles at 20,000 rpm for 3 min each).

#### 4.4.2. Droplet Size and Distribution

The droplet size distribution of the emulsions was determined using laser diffraction. The surface mean diameter (D [3,2]) was reported, with measurements taken at 25 ± 1 °C.

#### 4.4.3. Microstructure of Emulsions

Optical microscopy and confocal laser scanning microscopy (CLSM, TCS SP8, Wetzlar, Germany) were used to analyze the emulsion’s microstructure. The samples were stained with Nile Red (lipid phase) and FITC (aqueous phase) and observed using CLSM with 530 nm and 488 nm excitation wavelengths [52].

#### 4.4.4. Rheological Properties of Emulsions

The rheological properties of the RPSC emulsion were assessed as previously described for the RPSC gels.

#### 4.4.5. Emulsion Stability Tests

The stability of emulsions was assessed through centrifugal, freeze–thaw, and storage tests. For these tests, samples were respectively centrifuged (8000× *g*, 30 min); subjected to three freeze–thaw cycles (−20 °C/24 h to ambient temperature/4 h per cycle); and stored at 50 °C for 14 days. Changes in appearance, droplet size (D [3,2]), size distribution, and microstructure were evaluated after individual treatments and at intervals during storage (days 0, 3, 7, 14) [54].

### 4.5. Assessment of In Vitro Wound Healing Potential

#### 4.5.1. Cell Culture

3T3 cells were cultured in complete medium (37 °C, 5% CO_2_) and subcultured at 80% confluence. For experiments, cells were treated with RPSC at concentrations of 50, 100, 500, 1000, and 5000 μmol/L.

#### 4.5.2. Cell Viability

Cell viability was assessed by MTT assay [17]. Cells seeded in 96-well plates (5000 cells/well) were treated with RPSC for 24 h. Absorbance at 490 nm was measured using a microplate reader (Tecan, Männedorf, Switzerland).

#### 4.5.3. Cell Migration: Scratch Assay and Transwell Assay

Cell invasion and migration were assessed using Transwell chambers. The cells were seeded (5 × 10^3^ cells/well) in the upper chamber of the Transwell insert, while the lower chamber was filled with 800 μL of complete medium. After 24 h of incubation, the insert was removed, and non-migrated cells on the upper surface were gently wiped off using a cotton swab. The migrating cells were fixed with 4% paraformaldehyde and stained with 0.25% crystal violet solution for 20 min. Finally, the cells were observed under a microscope. The number of cells crossing the membrane was counted by ImageJ (version 1.8.0).

The scratch assay was performed to determine the migratory ability of 3T3 cells after co-culture with RPSC. Cells from each group were seeded in 6-well plates and incubated until they reached 80% confluence. A scratch wound was created in the cell monolayer using a 200 μL pipette tip, and the detached cells were washed away using phosphate-buffered saline (PBS). The cells were then cultured for additional 24 h. Cell migration was assessed by quantifying the wound closure area (percentage value) via ImageJ.

#### 4.5.4. Enzyme-Linked Immunosorbent Assay (ELISA)

Levels of SOD and TGF-β1 in cell supernatants were measured using commercial ELISA (Nanjing Jiancheng Bioengineering Institute, Nanjing, China) kits according to the manufacturers’ protocols. Cell supernatants were collected at 24 h after RPSC treatment.

#### 4.5.5. Western Blot

Total protein was extracted, and concentrations were determined by BCA assay [78]. Proteins were separated by SDS-PAGE, transferred to PVDF membranes, and blocked. Membranes were incubated overnight with primary antibodies (Collagen I and GAPDH), followed by incubation with corresponding secondary antibodies. Protein bands were visualized by ECL, and band intensities were quantified with ImageJ, with GAPDH as the loading control.

### 4.6. Statistical Analysis

All data are expressed as the mean of triplicates. Statistical significance (*p* < 0.05) was determined by ANOVA in SPSS (version 25.0.0), with graphs plotted using Origin 2018 and statistical analysis aided by GraphPad Prism (version 9.5.1).

## Figures and Tables

**Table 1:** Amino acid composition of RPSC.

Amino Acid	% of Total Protein
Aspartic acid	9.73
Threonine	3.95
Serine	4.83
Glutamic acid	13.52
Glycine	21.50
Alanine	6.09
Cystine	1.44
Valine	2.79
Prolin	7.09
Methionine	1.19
Isoleucine	2.31
Leucine	3.29
Tyrosine	1.56
Phenylalanine	1.65
Histidine	0.24
Lysine	3.75
Arginine	8.00
Hydroxyproline	6.20

## Data Availability

The data presented in this study are available on request from the corresponding authors.

## Abbreviations

The following abbreviations are used in this manuscript:

RPSC	Pepsin-soluble collagen derived from salt-preserved *Rhopilema esculentum*
β-me	β-mercaptoethanol
Td	Thermal denaturation temperature
SOD	Superoxide Dismutase
ROS	Reactive Oxygen Species
TGF-β1	Transforming Growth Factor-beta 1
Smad7	Mothers against decapentaplegic homolog 7
WB	Western Blot
MCT	Medium-chain triglycerides
SDS-PAGE	Sodium Dodecyl Sulfate–Polyacrylamide Gel Electrophoresis
CD	Circular Dichroism
DSC	Differential Scanning Calorimetry
SEM	Scanning Electron Microscope
CLSM	Confocal Laser Scanning Microscopy
FITC	Fluorescein Isothiocyanate
ELISA	Enzyme-Linked Immunosorbent Assay
BCA	Bicinchoninic Acid
PVDF	Polyvinylidene Difluoride
GAPDH	Glyceraldehyde-3-Phosphate Dehydrogenase
